# Glycosylation is a key in SARS-CoV-2 infection

**DOI:** 10.1007/s00109-021-02092-0

**Published:** 2021-05-22

**Authors:** Celso A. Reis, Rudolf Tauber, Véronique Blanchard

**Affiliations:** 1 i3S – Instituto de Investigação e Inovação em Saúde, Universidade do Porto, 4200-135 Porto, Portugal; 2 IPATIMUP – Institute of Molecular Pathology and Immunology, University of Porto, 4200-135 Porto, Portugal; 3 Instituto de Ciências Biomédicas Abel Salazar, University of Porto, 4050-313 Porto, Portugal; 4 Institute of Laboratory Medicine, Clinical Chemistry and Pathobiochemistry, Charité – Universitätsmedizin Berlin, corporate member of Freie Universität Berlin, Humboldt-Universität zu Berlin, and Berlin Institute of Health, Berlin, Germany

**Keywords:** SARS-CoV-2, Glycosylation, Spike protein, Blood group antigen, Infection, COVID-19

## Abstract

SARS-CoV-2 causes the respiratory syndrome COVID-19 and is responsible for the current pandemic. The S protein of SARS-CoV-2-mediating virus binding to target cells and subsequent viral uptake is extensively glycosylated. Here we focus on how glycosylation of both SARS-CoV-2 and target cells crucially impacts SARS-CoV-2 infection at different levels: (1) virus binding and entry to host cells, with glycosaminoglycans of host cells acting as a necessary co-factor for SARS-CoV-2 infection by interacting with the receptor-binding domain of the SARS-CoV-2 spike glycoprotein, (2) innate and adaptive immune response where glycosylation plays both a protective role and contributes to immune evasion by masking of viral polypeptide epitopes and may add to the cytokine cascade via non-fucosylated IgG, and (3) therapy and vaccination where a monoclonal antibody-neutralizing SARS-CoV-2 was shown to interact also with a distinct glycan epitope on the SARS-CoV-2 spike protein. These evidences highlight the importance of ensuring that glycans are considered when tackling this disease, particularly in the development of vaccines, therapeutic strategies and serological testing.

The SARS-CoV-2 causes the severe respiratory syndrome COVID-19 and is responsible for the current pandemic representing a significant threat to human health around the whole world [1]. At the moment of writing this manuscript, more than 234 million infections and three million of deaths have been reported worldwide. SARS-CoV-2 is an enveloped virus that belongs to the family of *Betacoronavirus* and the subfamily of *Sarbecoronavirus* [2] (Fig. 1). Its genome consisting of positive-sense single-stranded RNA shares over 96% whole-genome identity with the bat coronavirus RaTG13 and 90% with a pangolin coronavirus [1]. Moreover, SARS-CoV-2 is about 80% identical to SARS-CoV-1 and has about 50% identity with MERS-CoV that caused severe acute respiratory syndrome outbreaks during 2002-2003 and 2012, respectively [4]. The SARS-CoV-2 genome encodes four structural proteins, the spike (S) glycoprotein, the membrane (M) protein, the envelope (E) protein and the nucleocapsid (N) protein [4]. The S protein of SARS-CoV-2 is extensively glycosylated with each protomer of the transmembrane homotrimeric protein displaying 22 *N*-glycosylation sites and several *O*-glycosylation sites [5]. Similarly, the S protein of other coronaviruses (feline coronavirus, SARS-CoV and MERS-CoV) has been reported recently to be densely glycosylated with *N*-glycans [6, 7]. Studies on other coronaviruses indicate that also the M protein may be *N*-glycosylated and/or *O*-glycosylated [8].

**Fig. 1:**
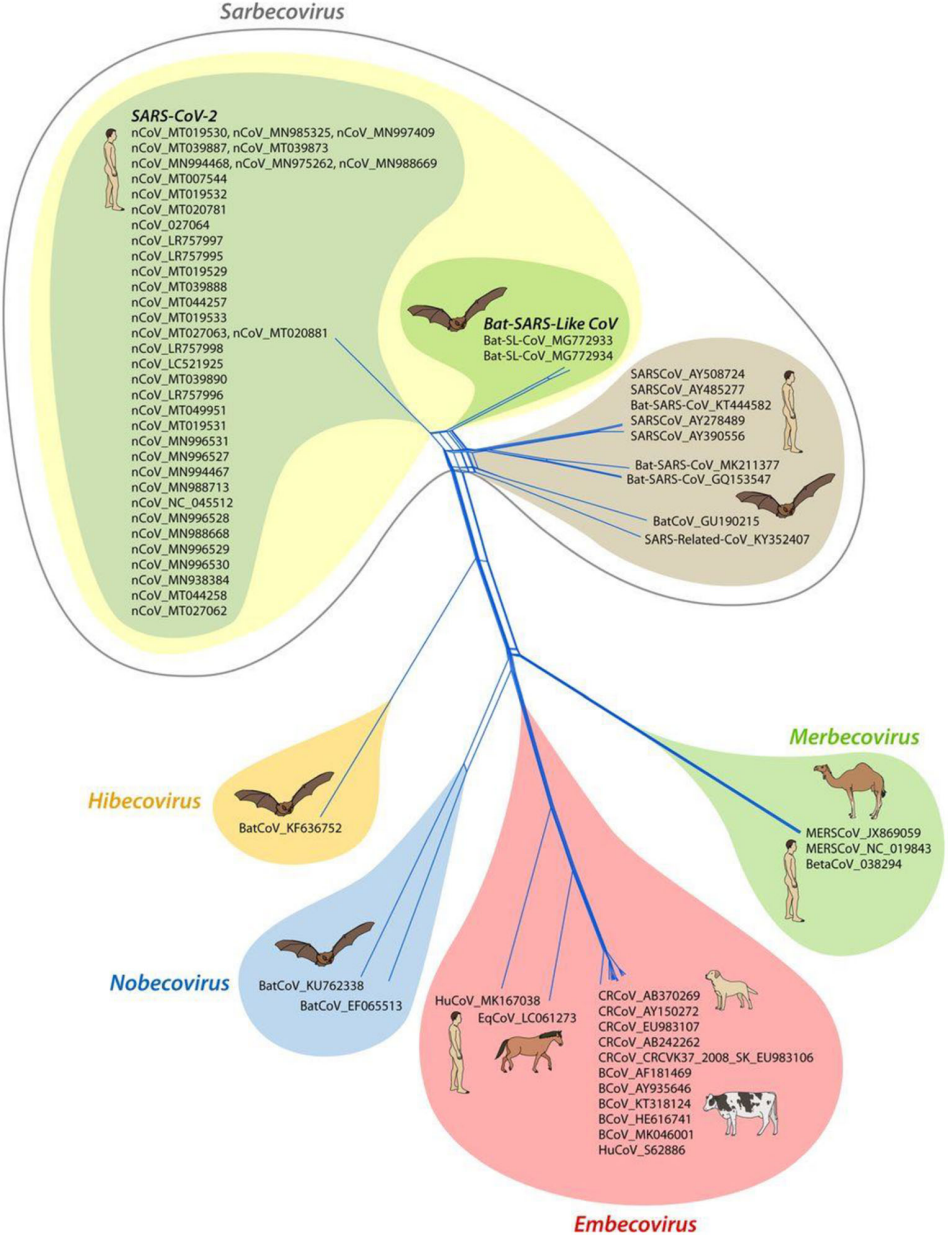
Spike (S) glycoprotein gene-based phylogenetic analysis. The analysis includes all five defined subgenera of Betacoronaviruses, namely, Sarbecovirus, Embecovirus, Merbecovirus, Nobecovirus and Hibecovirus. The isolates in the gray area are from the current outbreak of SARS-CoV-2 from around the world. The nearest neighbors of SARS-CoV-2 are the bat-SL-CoV, encircled in yellow (from [3] with permission)

Recent reports show that glycosylation of both virus and the target cells crucially affects SARS-CoV-2 infection at several levels: (i) virus replication and exocytosis, (ii) virus binding and entry to host cells, shaping viral tropism, (iii) innate and adaptive immune response, and (iv) therapy, vaccination and serological testing (Fig. 2).

**Fig. 2:**
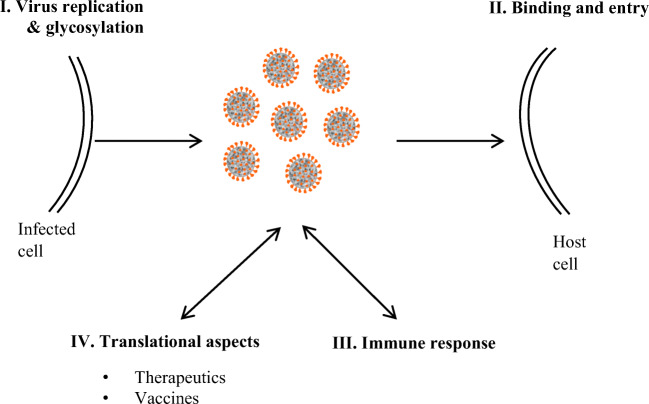
Role of glycosylation in SARS-CoV-2 biology and COVID-19 pathogenesis

## Virus glycosylation during replication and exocytosis

**Fig. 3:**
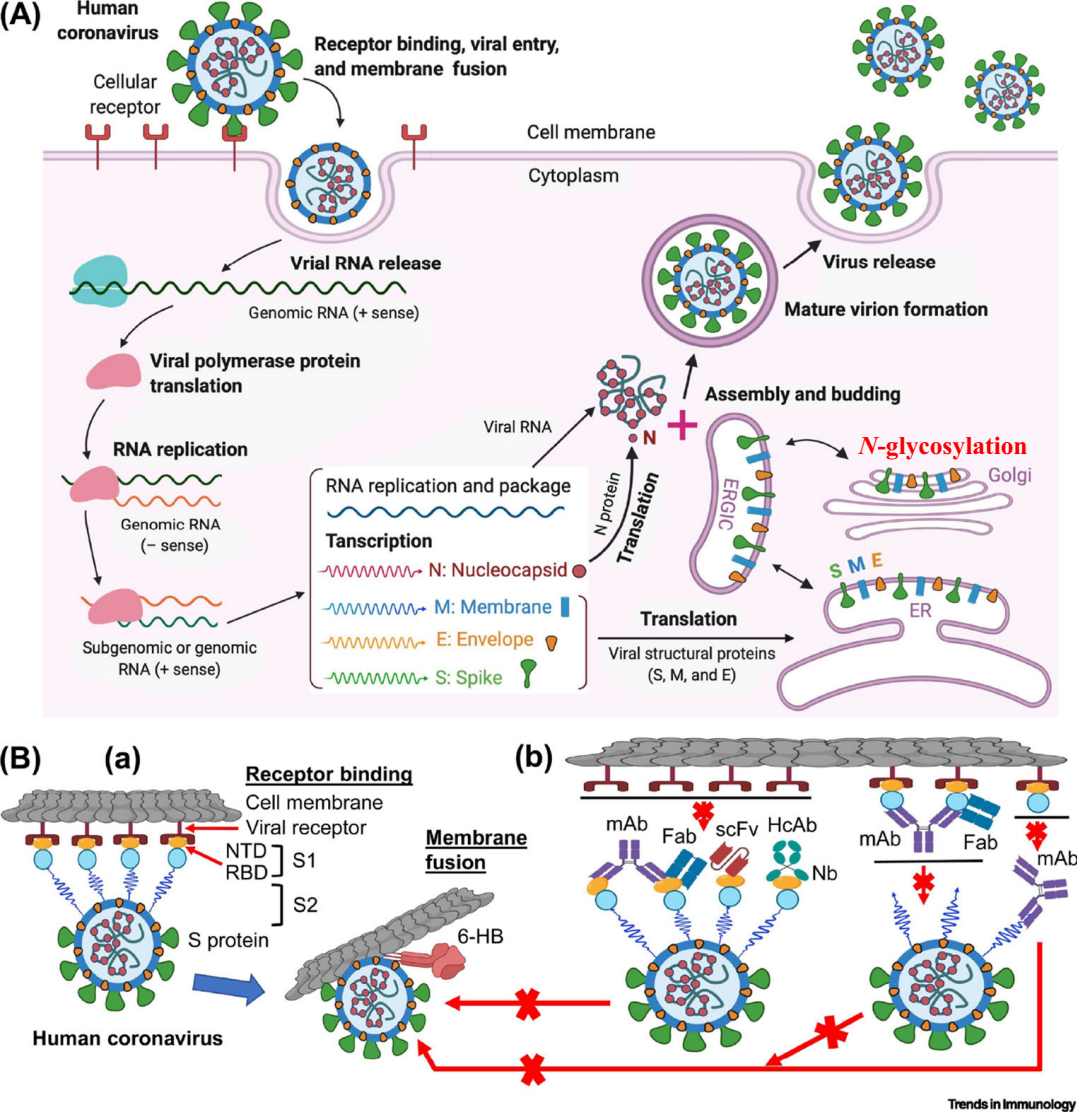
Steps of replication and subcellular site glycosylation of pathogenetic human coronaviruses (adapted from [10] with permission)

The replication mechanism of SARS-CoV-2 is currently studied in detail and appears to closely resemble the one of SARS-CoV and other coronaviruses [9]. SARS-CoV replication includes the formation of cytoplasmic replication complexes constituting of viral RNA and viral non-structural proteins and the transcription and translation of the four viral structural proteins that are translocated into the ER and transit to the ER-Golgi intermediate compartment (ERGIC), where encapsidated RNA virus particles are enveloped by viral transmembrane envelope proteins, envelope (E), membrane (M) and spike (S) [9]. Virus glycoproteins undergo *N*-glycosylation and *O*-glycosylation during transit in the ER, the ERGIC and the Golgi by the glycosylation machinery of the host (mammalian) cells (Fig. 3). Unlike mammalian glycoproteins, viral glycoproteins such as S protein contain a retrieval signal that retards their trafficking, thereby accumulating in the ER, ERGIC and Golgi where virus particles are assembled and then bud [11]. Viral glycoproteins undergo incomplete maturation, which results in higher levels of high-mannose *N*-glycans than those found in most mammalian glycoproteins [5].

The S protein of SARS-CoV-2 consists of an S1 subunit containing an *N*-terminal domain and the receptor-binding domain (RBD) and an S2 subunit membrane domain important for fusion of the virion with the host cell membrane. During biological processing and prior to budding, S1 und S2 subunits are cleaved via a furin cleavage site and S protein is assembled into a homotrimer [12]. Site-specific mass spectrometric analysis of the homotrimer after mutation of the furin cleavage site and expression in HEK293F cells revealed that all 22 *N*-glycosylation sites were occupied with oligomannose-type, hybrid-type or complex-type *N*-glycans [5, 13]. Overall, the extent of high mannosylation was about 30%, which is similar to that of the S protein from SARS-CoV but lower than those described for other viral glycoproteins such as HIV-1 envelope glycoprotein that consists of a 60% high-mannose *N*-glycans [6, 13]. Interestingly, in the homotrimer, the RBD located in S1 contains two *N*-glycosylation sites at N331 and at N343 that are occupied mostly by complex-type asialylated core-fucosylated *N*-glycans and also by hybrid structures at N343 [6, 13], suggesting that the immature nascent *N*-glycans at these two sites are accessible towards glycosidases and glycosyltransferases in the ER/Golgi. Interestingly, the biological processing of S1 and S2 was quite different when expressed as single domains independently: Only 17 *N*-glycosylation sites out of 22 were occupied; N331 and N343 were mostly occupied with high-mannose *N*-glycans and with asialylated core-fucosylated biantennary *N*-glycans [14]. The *O*-glycosylation sites in the RBD are probably not sterically accessible to GalNAc transferases in the nascent S trimer as they were only detected as traces in the homotrimer [5, 13]. The 5 *N*-glycosylation sites from the C terminus of S2, the domain responsible for invasion and fusion, are mostly occupied with complex-type glycans [6, 13]. Interestingly, traces of sulfation and diLacNAc motives were also observed on *N*-glycans of SARS-CoV-2 S protein [13], which are hallmarks of the HEK293F expression system but may also be glycoprotein-specific [15].

It is important to note that SARS-CoV-2 glycosylation does reflect the specific features of the glycosylation machinery of the host cell and will therefore vary with the cell type, where viral replication takes place. Moreover, since glycosylation exhibits also inter-individual differences, the viral glycosylation pattern may differ among patients infected with the virus (see the “Innate and adaptive immunity” section).

## Virus binding and entry (Fig. 4)

Glycosylation of the virus as well as of the host cells is centrally involved in SARS-CoV-2 binding and entry in at least four different ways. First, infection experiments employing primary human bronchial epithelial cells and authentic SARS-CoV-2 virus clearly demonstrated that the glycosaminoglycan heparan sulfate of the cellular glycocalyx is required to mediate infection of these target cells by SARS-CoV-2 [16]. Heparan sulfate was shown to interact with the receptor-binding domain of the SARS-CoV-2 spike glycoprotein, adjacent to ACE-2, shifting the spike structure to an open conformation to facilitate ACE-2 binding [16]. This finding extends the previous observation that heparin saccharides decrease the binding of the S protein to ACE-2 through interaction of the RBD of S with heparin saccharides [17].

**Fig. 4:**
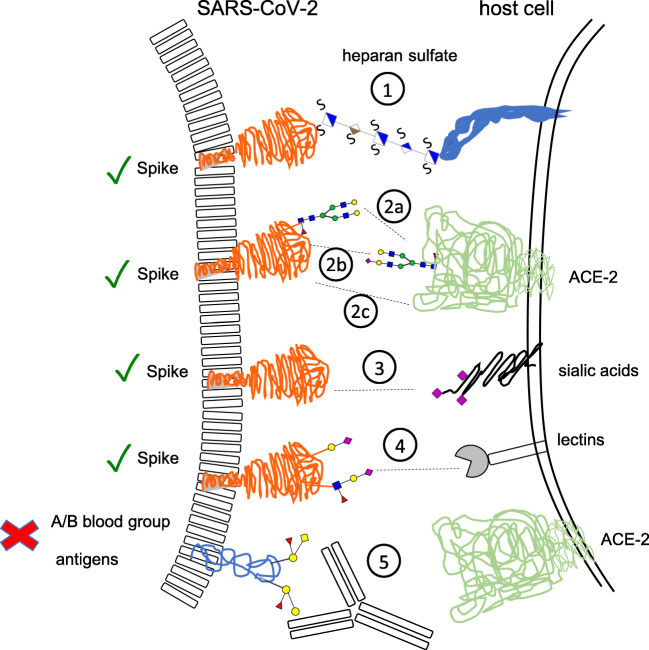
Proposed mechanisms of glycan-mediated host invasion of SARS-CoV-2. Receptor binding and invasion are facilitated by the interaction of its S protein with (1) heparan sulfate, ACE-2 receptors via (2a) glycan-glycan, (2b) glycan-protein, (2c) protein-protein interactions, (3) sialic acids and (4) lectins. In addition, A/B blood antigens (5) at the virion surface may prevent infection in a potential host producing blood group-specific antibodies. ✓ indicates inter-molecular interactions that promote receptor binding and invasion. X indicates glycan-mediated interactions that may prevent infection

Second, for entering host cells, SARS-CoV-2 utilizes the cell surface receptor ACE-2 as a point of entry as has been shown by cryo-electron microscopy, X-ray crystallography and cell culture experiments [12, 18–21]. The ACE-2 receptor is a membrane glycoprotein that is *N*-glycosylated as well [22]. Molecular dynamics simulations conducted to examine the co-complex of glycosylated S protein with glycosylated ACE-2 revealed glycan-protein and glycan-glycan interactions apart from protein-protein interactions. The sialylated complex-type glycans at N090 and N322 of ACE-2 were shown to form glycan-protein interactions with several regions of the S trimer [13]. In addition, complex-type and hybrid *N*-glycans at N546 of ACE-2 are in contact with the glycans at N0074 and N0165 located in the *N*-terminal domain of S [13]. Glycans at N165 and N234 of the S protein were shown to modulate the conformation of S protein’s receptor-binding domain [23].

Third, by contrast to SARS-CoV, S protein from SARS-CoV-2 was found to have a sialic acid binding pocket at its N-terminus that is similar to the one of MERS-CoV [24] indicating that sialic acids probably play a role in SARS-CoV-2 binding. Sialic acids are 9-carbon monosaccharides that terminate *N*-glycan, *O*-glycan and glycosphingolipid chains and occur at high abundance on the cellular glycocalyx.

Fourth, NMR studies showed that the two *N*-glycans of the RBD of the S protein at N331 and N343 bind to human lectins galectin-3, 7 and 8, Siglec-10, macrophage galactose lectin (MGL) and dendritic cell-specific intercellular adhesion molecule-3-grabbing non-integrin (DC-SIGN) [25]. Since these lectins recognize specific ligands in complex-type glycans, the findings indicate that additional mechanisms could be involved in the tropism and binding of SARS-CoV-2 to host cells. DC-SIGN was shown to mediate cell entry of SARS-CoV [26]. Galectin-1 was shown to stabilize HIV-1 attachment to host cells promoting HIV-1 infectivity [27].

## Innate and adaptive immunity

Glycans play an important role in innate and adaptive immunity and it early became clear that, in the SARS-CoV-2 infection context, they can have a sweet and sour role, i.e. either playing a protective role or contribute to immune evasion.

**Fig. 5:**
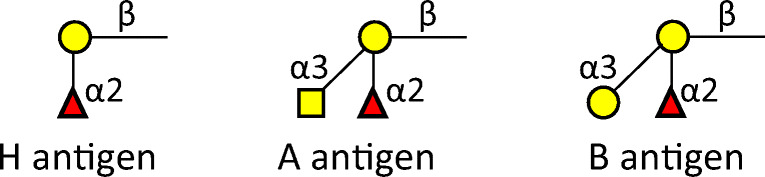
Human blood group antigens. Yellow circle, galactose; yellow square, *N*-acetyl galactosamine; red triangle, fucose

One of the most evident protective mechanisms is the potential immunity generated by the glycan determinants of the ABO histo-blood group antigens [28]. Studies in samples collected from blood donors in France revealed that the proportion of seropositives was significantly lower in group O donors when compared with other blood group donors, suggesting that blood group O individuals have lower risk of being infected [29]. In addition, genome-wide association studies in COVID-19 patients with severe disease in several hospitals in Italy and Spain identified a 3p21.31 gene cluster as a genetic susceptibility locus in patients with COVID-19 with respiratory failure. A blood group-specific analysis further showed a higher risk in blood group A than in other blood groups and a protective effect in blood group O as compared with other blood groups [30]. These observations are supported by a recent meta-analysis concluding that blood type A might be more susceptible to infect COVID-19 as compared to blood group O [31]. The reasons for the influence of ABO histo-blood groups remain to be unraveled. However, it is very likely that glycosylation of the S protein plays an important role, since, as outlined above, glycosylation of the S protein displays glycosylation features of the infected host cells i.e. of the infected individual. The synthesis of the ABO histo-blood group antigens is determined by distinct gene alleles at the ABO locus. In A and B blood groups, an N-acetylgalactosamine and a galactose, respectively, are transferred in an α1,3 linkage on the precursor H-type precursor structure, generating the corresponding A or B antigens (Fig. 5). The blood group O is determined by the O alleles, which are null alleles responsible for a lack of glycosyltransferase activity, and therefore the H antigen remains unmodified. The presence of O alleles in a homozygosity leads to the blood group O, characterized by the absence of A or B antigen expression. Blood group O individuals develop anti-A and anti-B antibodies induced by the exposure to the microbiota. Similarly, blood group A and B individuals develop either anti-B or anti-A antibodies, respectively [28]. It is therefore expected that when virions are produced in cells that express the enzymes responsible for the A or B blood group, they display the corresponding glycan antigen, and therefore in blood group O individuals, the anti-A and anti-B antibodies could prevent the infection [28]. Further studies addressing levels of anti-glycan antibodies (ABO or other) remain of potential great interest.

The glycosylation status of IgG at asparagine 297 also contributes to the cytokine storm described in severe COVID-19. The level of afucosylation at asparagine 297 of IgG antibodies directed against SARS-CoV-2 was particularly elevated in critically ill patients amplifying pro-inflammatory cytokine release and acute phase responses, but not in those with mild symptoms **[**32**]**. Additionally, glycans are ligands of endogenous lectins expressed by different immune cells and capable of regulating various functional aspects of innate and adaptive immunity [33]. As noted above, *N*-glycans of the RBD of the S protein were identified as binding partners of a wide range of human lectins involved in innate and adaptive immunity. Additionally, it is of interest to note that sulfated glycans found on the SARS-CoV-2 S protein may serve as selectin ligands [34] and may have a role in immune regulation.

Glycans can have major impact in sterically masking polypeptide epitopes in viral proteins with major consequences for immune evasion, for neutralizing antibodies and the design and efficacy of vaccines. Previous studies with other viruses showed that glycan density plays a role in shielding immunogenic epitopes. This mechanism of immune evasion has been well characterized for other viral glycoproteins, such as the HIV-1 envelope protein [35], the influenza hemagglutinin [36] and the Lassa virus glycoprotein complex [37]. Recent site-specific analyses of *N*-linked glycosylation on trimeric S proteins revealed that glycans contribute to the formation of a cluster of *N*-glycans at specific regions on MERS-CoV S [6]. Additional molecular evolution analysis of SARS and MERS S genes also showed a higher incidence of amino-acid diversity on exposed surfaces of the S protein that are not hidden by *N*-linked glycans [6]. Similar analysis to SARS-CoV-2 S variations may contribute to further elucidate the role of glycans in immune evasion, as well as neutralizing antibodies and the development of effective vaccine strategies.

With respect to the role glycans of both SARS-CoV-2 and host cells have in cell binding and immune response, variations in glycosylation of both virus and host are likely to influence tissue tropism and individual susceptibility to infection. As for the host, glycosylation may vary depending on cell type and tissue [38], age, ethnicity, individual expression profiles of glycosylation-related genes [28] and disease [38]. In addition, mutations of SARS-CoV-2 and ACE 2 leading to alterations of *N*-glycosylation sites could be responsible for differences in virus infectivity and patients’ susceptibility, respectively. The relevance of the observation that so far no variation of the glycosylation sites of SARS-CoV-2 has been observed during the global transmission course remains to be evaluated [39].

## Translational aspects

With respect to the role of SARS-CoV-2 glycosylation for virus replication, infectivity and immune response, glycosylation has major potential impact on therapeutic and vaccination strategies as well as on serological testing.

### Vaccination

Current vaccination strategies are based on the use of (a) structural immunogenic virus proteins, (b) attenuated or inactivated virus, (c) recombinant vector vaccines and (d) nucleic acid vaccines [40]. Glycosylation of SARS-CoV-2 will, however, differently impact each of these different strategies. The use of immunogenic structural virus proteins as vaccines is based on the production of recombinant proteins in prokaryotic or eukaryotic expression systems. Similar to the development of SARS-CoV vaccines [41], the heavily glycosylated SARS-CoV-2 S protein is a key target for the development of SARS-CoV-2 vaccines [42]. Glycosylation of the recombinant S glycoprotein does depend on the glycosylation machinery of the chosen expression system. For instance, the nanoparticle vaccine NVX-CoV2373 is composed of recombinant trimeric full-length SARS-CoV-2 spike glycoproteins expressed in the established baculovirus *Spodoptera frugiperda* (Sf9) insect cell expression system [42]. The majority of recombinant *N*-glycoproteins expressed in baculovirus-Sf9 insect cells are glycosylated with simple, non-sialylated, paucimannose glycans at sites that are glycosylated with complex-type, sialylated *N*-glycans in mammalian cells [43].

As may be inferred from studies obtained for other recombinant vaccine glycoproteins, glycosylation of SARS-CoV-2 vaccine spike glycoprotein may influence the immune response of the vaccinated individual in several ways:

1. As glycosylation, particularly *N*-linked glycosylation, profoundly affects protein folding and oligomerization during biosynthesis in the ER and the Golgi [44], it is very likely that proper folding of the immunogenic polypeptide epitopes of vaccine glycoproteins will at least partly depend on a suitable glycosylation of the recombinant protein. Moreover, in view of the dense glycosylation of the S protein, it is highly likely that glycans influence the epitope accessibility for antibodies targeting the S protein.
2. The presence of non-human glycans on recombinant therapeutic glycoproteins may result in the induction of antibodies directed against these glycan epitopes, in the clearance through pre-existing antibodies from serum and in the induction of IgE-mediated anaphylaxis [45].
3. Proper glycosylation of recombinant vaccine glycoprotein may influence the ability to raise an effective adaptive immune response, since modification of protein antigens by glycans influences cellular uptake, proteolytic processing, presentation by MHC and subsequent T-cell priming [46].

Whether antibody-dependent enhancement as observed with dengue virus, Zika virus, Ebola virus and coronaviruses involves glycosylation-dependent mechanisms remains to be studied [47]. In conclusion, appropriate glycosylation of SARS-CoV-2 antigens should be taken into consideration for the development of effective prophylactic vaccines.

### Therapy

In view of the role for binding and entry into the host cell, the spike protein of SARS-CoV-2 as well as the ACE-2 receptor is a potential target for inhibition by small molecules or antibodies that might block host receptor binding and/or membrane fusion [37]. Interaction of both spike protein and ACE-2 receptor with inhibitory small and large molecules will be influenced by glycosylation. Pinto and coworkers demonstrated that a human monoclonal antibody generated against the RBD of S from SARS-CoV was also able to neutralize SARS-CoV-2 [48]. Remarkably, this human monoclonal antibody does not only bind to amino acids of the RBD region of S, but it also interacts with the core fucose of the *N*-glycan at N343 in SARS-CoV-2, which corresponds to N330 in SARS-CoV [48]. When designing therapeutic antibodies directed against the S protein, the shielding of relevant epitopes by S protein’s glycans must be considered. Moreover, since the *N*-glycan at N297 of IgG strongly influences binding to Fcγ receptors [49], the glycosylation of biotechnologically produced therapeutic antibodies will have to be designed precisely to consider this issue.

### Serological testing

The detection of anti-SARS-CoV-2 antibodies using ELISA or related technologies employs recombinant structural SARS-CoV-2 proteins as antigens. Avidity of the antigen/antibody interaction determined by the binding affinity of the proteins, the charge and the structural arrangement of the proteins in the complex, may likely be influenced by glycosylation [50]. To ensure comparability of test results even when using the same test, care must be taken regarding glycosylation of the recombinant glycoproteins used that is kept constant from batch to batch. Further studies addressing these issues may provide crucial information on the glycosylation impact regarding serological assays’ specificities and sensitivities.

In view of the importance of glycosylation for therapeutic proteins, for vaccination and for serological testing, the design and the analysis of glycosylation are of central significance for the production of recombinant glycoproteins used for vaccination, therapy or serological testing. Glycan profiling will be significant not only for the design of effective glycoproteins, but also for quality control of the manufacturing processes.

In summary, being an enveloped virus, SARS-CoV-2 envelope proteins display the glycans that are produced in the infected cells. As described above, the glycosylation of a given cell is dependent on the expression of glycosyltransferases, which are differentially expressed between cell types within the same individual, as well as present variations from individual to individual. These are important issues with biological and immunological implications. A large set of data is pointing towards the key role that glycans have in several aspects of the SARS-CoV-2 infection, COVID-19 disease progression and clinical approaches. These evidences highlight the importance of ensuring that glycans are considered when tackling this disease, particularly in the development of vaccines, therapeutic strategies and serological testing.
